# Angela Merkel’s Leadership Lessons: the Secret of Political Longevity (2013‒2021)

**DOI:** 10.1134/S1019331622080111

**Published:** 2022-06-29

**Authors:** E. P. Timoshenkova

**Affiliations:** Institute of Europe, Russian Academy of Sciences, 125009 Moscow, Russia

**Keywords:** leadership, power, elections, chancellor, FRG, A. Merkel, “people’s parties,” opposition, migration crisis, COVID-19, success rhetoric, democracy, image

## Abstract

The article examines the phenomenon of the political longevity of German Chancellor Angela Merkel. The subject of the study is the second period of her chancellorship (2013–2021), as few German leaders have managed to stay in office longer than two terms. The author analyses in detail what objective factors and personal qualities helped Merkel to win the 2013 and 2017 elections, overcome declining personal popularity and objective voter fatigue, and get through all crises, including the migration crisis and COVID-19. The article examines how the tactics and strategy of the first female chancellor have transformed in response to changing political conditions. It explores the tools with which Merkel built her image as nonpartisan leader of the nation, and how she became a hostage to the image she created in 2015. The decision to accept refugees was a turning point in Merkel’s career; thus, the author pays particular attention to the ways in which she helped maintain her chancellorship. The paper uses the theory of how women’s leadership differs from men’s. The conclusion is drawn that Merkel developed her own special personal method of securing power and maintaining the image she needs. The main secret of her success is not the use of force but quick learning and adaptation, based on her instinct for political survival.

Angela Merkel has the ability to extend her political lifespan. She headed the German government for 16 years from 2005 to 2021. Before her, only Helmut Kohl, who entered the history of Germany as the unifying chancellor of the country, had been at the top of power for so long. Four times German citizens voted for Merkel and the CDU. At favorable moments in her political career, three words—“you know me”—were enough for her to win the elections. In difficult times, one phrase—“we will manage”—was enough to split German society and cause hatred for herself and the migration course among a significant part of the citizens.

According to Joschka Fischer, the former of Foreign Affairs of Gerhard Schroeder’s red-green cabinet (1998–2005), Merkel spoiled her “rendezvous with history” with the beginning of the euro crisis.^2^ However, during her tenure as chancellor of Germany, Merkel, unlike her predecessors, had not one but several significant “meetings with history”: the banking crisis of 2008, the euro crisis of 2010, the migration events of 2015, and the COVID-19 pandemic. Each time it was about specific problems that required quick and far-reaching strategic decisions in the absence of ready-made recipes. All these dramas took place not at the level of a nation-state but in the world arena. There were no proven tools that politicians could use. In fact, Merkel had to become a “politician-fireman.”

In November 2015, in a speech dedicated to the memory of German Chancellor H. Schmidt (SPD), Merkel noted that “the achievements of this politician were especially noticeable in the way he dealt with crises” (Bollmann, 2021, p. 11). This criterion applies to Merkel herself. International crises changed not only the chancellor’s approach to governing the country, her style, and original goals, but also Germany’s position in the world. For many decades, the German government has sought not to be left alone in the international arena. However, the euro crisis, which revealed the economic weakness of France; the exit of Britain from the EU; the US refusal to lead the military-moral role in the West, which was a gradual process under the presidency of B. Obama and devastating under D. Trump; and the danger of war in Ukraine brought Germany to a new level of political influence, increasing its importance in Europe and the world. It was Merkel’s image—a modest and open leader, willing to compromise and firmly on her feet—that helped Germany’s neighbors to accept its new role. Under the circumstances, the chancellor managed to achieve the maximum possible for her country, while using a minimum of external domination.

Foreign observers began to analyze the phenomenon of Merkel’s longevity earlier than German political scientists and more closely (Florence, 2017; Braun, 2015; Bunelli, 2013; Gastronovo, 2014; Grawford and Czuczka, 2013; Qvortrup, 2017; Renterghem, 2021; Schramm, 2016) than their colleagues, who probably believed that they had studied the chancellor thoroughly by the third election period (Bollmann, 2013; Meng, 2006; Heckel, 2009; Languth, 2005; Mishra, 2010; Stock, 2005). However, the policy of “open doors” for migrants in 2015 turned out to be unexpected for many experts. A significant part of recent research in Germany falls on this period and is devoted to the analysis of the reasons that prompted Merkel to accept a huge flow of refugees, as well as the transformation of her leadership after these events (Alexander, 2017; Knaus, 2020; Münkler H. and Münkler M., 2016). In Russian political science, two scientific centers can be distinguished that consistently analyze Merkel’s domestic and foreign policy: the Institute of Europe of the Russian Academy of Sciences and the Institute of World Economy and International Relations of the Russian Academy of Sciences (*Sovremennaya Germaniya …*, 2015; German Economy and Politics …, 2019; Timoshenkova, 2020; Vasil’ev, 2018; Vasil’ev, 2021; Kokeev, 2021; Horol’skaya, 2021; Timofeev and Horol’skaya, 2021; Basov, 2019). Special attention needs to be paid to the works of N.V. Pavlov, Dr. Sci. (Hist.), and A.A. Derevyanchenko (Pavlov, 2018a; Pavlov, 2018b; Pavlov, 2019; Derevyanchenko, 2017).

The controversial decision in 2015 led Merkel to her gradual loss of power and popularity. After the forced repetition of the “grand coalition” following the results of the election to the Bundestag in 2017 and the voluntary resignation of the CDU chair (2018), many political scientists predicted her early departure from the post of chancellor (Alexander, 2021; Hebel, 2018; Levitsky and Ziblatt, 2018).

The weakening of the power resource and the promise to leave politics at the end of the fourth term of office led to the fact that, on the one hand, Merkel turned into a “lame duck,” and on the other hand, the accumulated international prestige and the lack of need to compete for the fifth time gave her more freedom of action. It is no coincidence that in 2017, after the unpredictable Donald Trump was elected president in the United States, the world community recognized the German chancellor as the main defender of democracy and Western values.^3^

This article analyzes the methods of securing the chancellor on the political Olympus of Germany, the transformation of her image and decisions that contributed to the preservation of power over the last legislative periods. Merkel’s foreign policy activities are not included in the study, as they are a separate topic for investigation.

## MERKEL: A HOSTAGE TO HER OWN MODEL OF SUCCESS (2013–2017)

The 2013 elections were the pinnacle of success for Merkel and the CDU. Together with the CSU, the Christian Democrats received a record 41.5% of the vote. This election campaign, like the previous one, relied on the chancellor’s image as an experienced and predictable politician. Merkel was convinced that the main condition for winning the elections was the calmness of citizens and their confidence in the future. Therefore, her main task was to minimize risks and maintain what had already been achieved. Merkel’s principle—“you know me”—worked perfectly. However, the strong results of the CDU/CSU were not enough to create a government majority. The FDP failed to overcome the 5% electoral threshold and did not enter the Bundestag for the first time in history. Staying in the government under the leadership of strong Merkel did not allow her to fulfill her election promises, and the tactics of hard pressure on the chancellor, which were used by the liberals, contributed to the creation of an image of an unreliable and scandalous partner. As a result, the CDU/CSU again formed a “grand coalition” with the SPD.

Thus, during the first period of her chancellorship (2005–2013), Merkel perfectly mastered the techniques of “asymmetric demobilization” and “soft absorption” of competitors (Schumacher, 2006, p. 71). The former involves the adaptation of the main election slogans of the rivals and their introduction into the program of the CDU. The latter is characterized by a tactical way out of situations. Merkel can be compared to a sumo wrestler who does not throw his opponent to the ground with force, but dodges him so skillfully that an energetic opponent falls by himself. Observers and participants in the political process often interpreted her restraint and avoidance of open conflict as inaction (Blome, 2013, p. 10). Therefore, the victories looked unexpected.

A minimum of risk and reasons for criticism is a principle that Merkel successfully used in the elections and firmly embedded in domestic politics. She tried to get away from public discussion not only of sensitive topics but also of those that could contain conflict potential. For example, the chancellor rarely visited the German troops and police, although she regularly invited their representatives to meetings. At the same time, she avoided being photographed against the background of military equipment and always emphasized that the main goal of these structures is to maintain peace and order (Alexander, 2018, p. 17). Another sensitive topic was migration.

In mid-2014, Merkel’s trusted collaborator Eva Christiansen—head of the “political planning, major issues, and special assignments” headquarters—brought in three specialists in the psychology of collective behavior to develop recommendations on how best to interact with citizens for their own good. As a result, the concept of “civil dialogue” emerged, the main motto of which was “it is good to live in Germany” (Alexander, 2018, p. 25). This was a wide-ranging attempt by the chancellor’s office to present Merkel as a nonpartisan leader and the “mother of the German nation,” who cares about her citizens and was sympathetic and aware of all problems, no matter how big.

Merkel’s success in winning the sympathy of society and demobilizing competitors was so convincing that in June 2015 the Social Democrats seriously discussed the possibility of refusing to nominate their candidate for the post of chancellor in the next election, because they saw no chance of winning (Bannas, 2019, p. 46). The situation changed dramatically in September. Merkel from the most popular politician instantly turned into the most controversial political figure in Germany. Researchers are still wondering what made the chancellor open the state borders to more than a million refugees and immigrants, among whom the proportion of those who fled the war in Syria and were eligible for political asylum was insignificant.^4^ There are various explanations, including the ones given below: Merkel wished to go down in history as a great humanist and rescuer; she was guided by economic calculations, since the German market economy needed an influx of foreign labor; she hoped to improve the image of Germany, which unleashed two world wars; her Christian upbringing and female empathy played a role; and the chancellor turned out to be short-sighted and did not understand at all what consequences her decision would lead to (Resing, 2017, p. 123; Knaus, 2020, p. 7).

There is another noteworthy version, according to which Merkel was a hostage to her own principles and management style. After becoming chancellor of the Federal Republic of Germany, for a long time she diligently avoided publicly expressing her attitude on the topic of migration. Nevertheless, on October 31, 2014, on Reformation Day, during a speech at the Church of Mary Magdalene in Templin (Brandenburg), Merkel had to answer the question of how Christian policy correlated with the expulsion of immigrant families well integrated into German society. According to the chancellor, such a measure was not Christian only at first glance; and it would be even worse to accept many, thereby depriving those who really needed protection (Alexander, 2018, pp. 27, 29).

The onset of the 2015 migration crisis did not affect Merkel’s strategy. She still tried to distance herself as much as possible from this topic, resisting pressure from the public and the media that demanded that she voice her attitude and at least visit a center for migrants. In the summer of 2015, German President Joachim Gauck met the refugees, and Foreign Minister Frank-Walter Steinmeier (SPD) and Labor Minister Andrea Nahles (SPD) addressed this topic. The chancellor remained silent. She explained her inaction in a private conversation as follows: “I was elected chancellor to solve problems. If I go there (meaning, the refugee center—*Author*), then I must have a solution” (Alexander, 2018, p. 29).

However, on July 15, 2015, while visiting a school in Rostock, she found herself in a difficult situation right in front of journalists’ cameras. A 14-year-old girl, who was born in a Palestinian refugee camp, admitted with tears that her biggest fear was leaving Germany because her family had no right to stay. Thus, Merkel found herself in a dilemma: to maintain the image of a caring and warm-hearted woman, on which she had been hard at work lately, or to remain head of government and side with the officials who made this decision. At first, she tried to calm the girl. However, then she answered: “If we now say that you can all come, and you really will all come from Africa, then we risk not being able to cope with this” (Alexander, 2018, p. 31). The footage of the conversation was covered by many media outlets. The chancellor was accused of coldness and cynicism not only by domestic but also by foreign, publications. The video of the scene became the most viewed on the Internet. *Stern* magazine called Merkel a “cold queen.”^5^ Her honest reaction to the girl’s words threatened to turn into an image disaster and break two images at once: a competent chancellor who always has a solution and a caring, humane leader.

During her time in government, Merkel became accustomed to start each week by analyzing statistics about what concerned German society. This helped her make decisions and respond to events in time. After the incident at the meeting with schoolchildren, 81% of the Germans surveyed were confident in the emotional coldness of the chancellor (Alexander, 2018, p. 33). At the same time, cases of attacks on refugee camps became more frequent in the country, which were used not only by the opposition but also by Vice-Chancellor Z. Gabriel for their own purposes. Society and politicians insistently demanded that Merkel break the silence and express her opinion. The decision of the chancellor to open the borders for refugees in late August–early September 2015 was influenced by the following factors: the speed of the problem, huge public pressure, lack of a unified position in the government, and fear of losing credibility and voter support.

The consequences of this decision changed Germany and split German society. The growth of radical right-wing sentiments, huge demonstrations against migrants, the problems of integration of refugees, the stunning rise of the Alternative for Germany party, the failure of the CDU in the land elections, the loss of the personal authority of the chancellor, and the threat of a constructive no-confidence vote from their own associates led to the fact that already in December 2016, Merkel officially stated at the CDU congress: “The situation at the end of the summer of 2015 should not be repeated. This grave humanitarian situation could only be overcome and brought under control by the methods that we applied. 2015 will forever remain an outstanding achievement of our country.”^6^

## LAST TRIALS (2017‒2021)

The leitmotif of the 2017 election campaign was Merkel’s promise that “Germany’s great achievement of 2015 will not be repeated.” The flow of refugees decreased significantly as a result of the measures taken by the government. The chancellor survived this crisis and won the election again. In fact, her decision to run for a fourth time was not dictated by a desire for change but by a desire to consolidate what had been achieved. Her reelection was not a triumph, and the formation of a government coalition was more difficult than ever in German history. It took Merkel about six months to form a government.^7^ At the same time, the first round of negotiations, in which the CDU/CSU, Union 90/Greens, and the FDP participated, failed. As the leader in the EU, Germany could not afford to be distracted by internal affairs for long. New elections would most likely bring even more votes to the right-wing radical Alternative for Germany (AfD) party, which Merkel recognized the day after the victory as the “main challenge” of her fourth chancellorship. The formation of a minority government would have made it more difficult for the Bundestag to pass laws and therefore govern the country. However, none of the potential partner parties was in a hurry to help the chancellor in this difficult situation. Liberals and Social Democrats saw firsthand that participation in government coalitions had led to the weakening of their parties and the loss of voters’ confidence. In the then situation, Merkel was largely to blame herself. On the one hand, her actions as the CDU chairman to conquer the political center of society and the “no alternative” to the chancellor’s course contributed to the creation of the AfD on the right flank, which took away votes from traditional parties. On the other hand, Merkel’s management style in coalitions destabilized her partners, who feared a repetition of negative consequences. The situation was saved by President of Germany Steinmeier, putting pressure on the SPD. As a result, the chancellor again led the “grand coalition,” torn apart by contradictions from all sides, including from her main ally, the CSU.

The crisis state of the government, the losses of the Christian Democrats in the land elections and the fall of their own authority in the party forced Merkel to announce on October 29, 2018, her voluntary resignation from the post of the CDU chairman. Thus, she violated her basic principle of maintaining leadership—the concentration in one hand of the leadership of the party and government. According to Merkel, it was the degradation of the power of her first rival Schroeder, which became a serious lesson for her (Blome, 2013, p. 108). Unlike her predecessor, the chancellor never risked her powers and did not succumb to public pressure, regardless of the level of personal ratings and party results. However, she decided to sacrifice less for the sake of saving more in this situation. Merkel remained true to her political survival instinct. At the same time, the chancellor promised to leave politics in 2021 and not run again. The actions taken allowed her to strengthen her authority and reduce the intensity of tension. In July 2018, 78% of the German population were dissatisfied with the work of the government, and Merkel’s personal trust rating was 48%, but by December 2018, this figure rose to 57%.^8^

The decrease in the authority of the chancellor was reflected in the behavior of her associates. Thus, to strengthen his own image, the CSU leader and Prime Minister of Bavaria Markus Söder avoided joint photographs with Merkel (Bollmann, 2021, p. 681). Proximity to her views, political course, and style of management turned into a controversial bonus for her successors, who needed to distance themselves from the chancellor and at the same time maintain the stability of the system. The situation with the coronavirus helped Merkel to strengthen her leadership and regain the trust of the German citizens. She was always perceived as a good crisis manager. The fight against the pandemic allowed her to show her strengths: outward calm, as well as scientific and at the same time humane approach to solving problems. Merkel’s speech to the deputies of the Bundestag during the second wave of the disease on September 30, 2020, was one of the best in her career. In it, the chancellor not only used well-developed and polished rhetoric techniques (imperative, warning, and personal empathy, emotionality combined with pragmatism), but also demonstrated her understanding of politics: she proposed guidelines, and civil society followed them. The key mechanism of such interaction is the responsibility of everyone. Merkel had not been so emotional and convincing since 2000—a speech at the CDU congress, in which she called for the revival of the party after the departure of Kohl.

Because all rules, regulations, all measures are useless if they are not accepted and observed by people. Therefore, we must speak … words that will reach as many people as possible …. We must all explain the danger and draw attention to the difficult situation that the cold season brings with it …. I think we all want to live the life that we knew …. But at the moment we are risking everything …. We must not allow … a dying person to be forced to die all alone in a hospital or in a nursing home, because people who love him cannot say goodbye to him, cannot extend a hand to him.^9^

The historical paradox lies in the fact that in her first important speech, which raised her to the political Olympus, Merkel operated on the rights and freedoms of citizens, and at the end of her reign she was forced to justify the need for their restriction. German voters positively assessed the government’s actions to combat the coronavirus and the personal contribution of the chancellor. Despite some costs and criticism of Merkel’s political management, 84% of citizens were confident in March 2021 that the chancellor would cope with the crisis and remain in office until the end of her term. In July, her personal trust rating rose to 83%, the best among German politicians.^10^ Thus, Merkel managed to overcome all the internal political crises that fell on her 16-year period of chancellorship and remain the number one politician in Germany, despite all the difficulties of the fourth legislative period.

## CONCLUSIONS

The scale of a politician is determined by the extent to which he/she corresponds to his/her time. During the chancellorship of Merkel, the familiar and stable world began to change rapidly. In foreign policy, the transformation of the postwar structure began; in domestic policy, the pandemic had an unexpected impact on the usual way of life. Having survived the collapse of the GDR in 1989/1990, she was probably better prepared than other Western colleagues for modern challenges.

Merkel began to lead the country as a “chancellor of change,” but quickly realized that the conservative German voters were afraid of drastic changes. Therefore, she tried not to impose radical reforms on them. The exception was the abolition of compulsory military service and the rejection of nuclear energy. Yet even here, she acted rather forcedly, following the moods of her citizens. This harmony ended in 2015 with the admission of a large number of refugees. Merkel could no longer protect Germany from world problems and entered a conflict the core of which was the choice between national security interests and openness to the world.

Merkel’s main principles in politics were pragmatism and minimum risk. She tried not to associate the political projects of the government with personal authority. Her main strategy is “asymmetric demobilization,” the goal of which is to form positive associations among voters. She turned this skill into a serious weapon against the opposition. Merkel strove to avoid criticism and direct clashes. Even being on the verge of impeachment, she did not dare to use her “chancellor’s competence” and dismiss the obstinate Minister of the Interior Horst Seehofer in 2018. In fact, Kohl turned out to be the only politician in her career against whom she spoke out openly and decisively.^11^ Regardless of the motives, this decision turned out to be justified for Merkel and ensured her career take-off. She fought with other rivals within her own party carefully and quietly. She sent some, like Federal Minister for the Environment Norbert Röttgen in 2012, to conquer the state election. It gave others the opportunity to subdue their ardor in the ministerial field: for example, the two main critics of her political course, Seehofer and Jens Spahn, received in 2017 the portfolios of ministers of the interior and health, respectively. Decisions that she could not influence, she presented as consensual and approved by her personally. Although from the outside, such actions can be interpreted as concessions and a sign of weakness, the example of Merkel proves that in a modern democratic society the secret of “political longevity” lies not in the use of force and “pushing through” the positions of others but in the ability to use forced retreats for one’s own purposes, find advantages in them, and turn them into successful strategies.

Merkel’s ability to pass off the merits and results of coalition governments as her own decisions and achievements seriously weakened her partners, while at the same time helping to strengthen her authority. The results of the election campaigns show that she managed to stay in office, despite significant losses and defeats of the CDU. This is contrary to the popular belief that voters perceive parties and candidates in aggregate. Merkel managed to create an image of a nonpartisan politician thanks to effective moderation and the ability to compromise. The decisive factor in retaining power was her leadership style, which guaranteed voters that she would definitely find the right solution and come to a consensus.

During the 16 years of chancellorship, Merkel’s image underwent a major transformation. She was called a “political killer,” compared with Margaret Thatcher; considered a follower; accused of lack of goals; admired for her ability to find compromises and get through crises; and recognized as a chancellor with a “firm hand” and a pragmatist who firmly stood on the ground. One constant was her ability to change quickly. Learning and a scientific-pragmatic approach had become the main secret of her success. Politician Merkel won all these years because she effectively applied Darwin’s theory of evolution to political processes, according to which survival is not about strength but about adaptability.

## Footnotes

2 Ex-Minister of Foreign Affairs: Joschka Fischer: Merkel has blown it, May 21, 2010. https://www.sueddeutsche.de/politik/ex-aussenminister-ueber-die-kanzlerin-joschka-fischer-merkel-hat-s-versemmelt-1.947223. Cited October 15, 2021.

3 Angela Merkel is now the leader of the free world, not Donald Trump. Sunny Hundal, February 1, 2017. https://www.independent.co.uk/voices/angela-merkel-donald-trump-democracy-freedom-of-press-a7556986.html; Harvard. Merkel-Rede gegen Trump. Deutschlandfunk. May 31, 2019. https://www.deutschlandfunk.de/harvard-merkel-rede-gegen-trump-100.html. Cited October 15, 2021.

4 In 2015, Germany was forced to take in 1.1 million refugees during the year, of which 476,649 applied for asylum. For a more detailed analysis of Merkel’s migration policy, see: Germany, 2015 (2016).

5 Die Eiskönigin—wie Angela Merkel zur meistgefürchteten Frau Europas wurde. Unser Thema im aktuellen stern. /jr http://t.co/uFwjryeCG6 (twitter.com); Stern punktet mit “Eiskönigin” Merkel, Spiegel mit “Versichert und verraten.” https://meedia.de/2015/08/18/stern-punktet-mit-eiskoenigin-merkel-spiegel-mit-versichert-und-verraten/. Cited October 15, 2021.

6 CDU-Parteitag: Rede von Angela Merkel am 06.12.2016. https://www.youtube.com/watch?v=rUqZHSK7Rt4; CDU-Parteitag: Versöhnung mit Schönheitsfehler, 6.12.2016, https://www.zeit.de/politik/deutschland/2016-12/cdu-parteitag-angela-merkel-rede-wahl?utm_referrer=https%3A%2F%2Fwww.google.de%2F. Cited October 15, 2021.

7 As a result of the election on September 24, 2017, the CDU/CSU received 37.2% of the votes, of which the CDU got 30.2%, and the CSU, 7%. The coalition agreement between the CDU/CSU and the SPD was signed on March 12, 2018.

8 ARD-DeutschlandTrend. https://www.tagesschau.de/inland/ deutschlandtrend/deutschlandtrend-1279.html; Infografik: (Un)zufrieden mit der Kanzlerin? https://de.statista.com/infografik/14253/ politikerzufriedenheit-angela-merkel/. Cited July 20, 2021.

9 Cited from: Frank Hartmann, Merkеls Rhetorik: Worte auf der Goldwaage. https://www.politik-kommunikation.de/ressorts/ artikel/worte-auf-der-goldwaage-363547423. Cited July 20, 2021.

10 Umfrage zum Verbleib von Angela Merkel als Bundeskanzlerin bis September 2021. https://de.statista.com/statistik/daten/ studie/1224901/umfrage/umfrage-zum-verbleib-von-angela-merkel-als-bundeskanzlerin/. Cited July 20, 2021.

11 This refers to Merkel’s open letter to *Frankfurter Allgemeine Zeitung* in 1999 and her speech at the CDU congress on April 10, 2000, in which she called for the renewal of the party after accusing its chairman Kohl of corruption—hiding the sources of funding for the CDU. (*Author’s note*).

## References

[1] Alexander R. (2017). *Die Getriebenen: Merkel und Flüchtlingspolitik: Report aus dem Inneren der Macht*.

[2] Alexander R. (2021). *Machtverfall: Merkels Ende und das Drama der deutschen Politik*.

[3] Bannas G. (2019). *Machtverschiebung. Wie die Berliner Republik unsere Politik verändert hat*.

[4] Basov, F. (2019), Elections of the CDU leader as the most important event in the process of power transfer in Germany, in *Doklady Instituta Evropy,* No. 360: *Ekonomika i politika Germanii: cherez god posle vyborov* [Reports of the Institute of Europe, No. 360: Economy and Politics of Germany: A Year after the Election], Ed. by E. P. Timoshenkova, Moscow: IE RAN, pp. 56–62.

[5] Blome N. (2013). *Angela Merkel: Die Zauder-Künstlerin*.

[6] Bollmann R. (2013). *Die Deutsche: Angela Merkel und wir*.

[7] Bollmann R. (2021). *Angela Merkel: Die Kanzlerin und ihre Zeit*.

[8] Braun M. (2015). *Mutti. Angela Merkel spiegate agli italiani*.

[9] Bunelli, R. (2013), *Angela Merkel, La sfinge,* Reggio Emilia.

[10] Derevyanchenko A.A. (2017). *Tri zhizni A. Merkel’: obychnaya, propedevticheskaya, triumfal’naya. Politicheskaya i lichnaya biografiya pervoj zhenshchiny–federal’nogo kanclera*.

[11] Timoshenkova E. P. (2019). *Doklady Instituta Evropy,* No. 360: *Ekonomika i politika Germanii: cherez god posle vyborov*.

[12] Florence, A. (2017), *Angela Merkel: Une Allmande comme les autres,* Paris.

[13] Gastronovo, V. (2014), *La syndrome tedesca: Europa 1989–2014,* Bari.

[14] Belov V. B. (2016). *Chast’ II. Doklady Instituta Evropy*, No. 328.

[15] *Germaniya. Vyzovy XXI veka* [Germany: Challenges of the 21st century] (2009), Ed. by V. B. Belov, Moscow: IE RAN, Ves’ Mir.

[16] Grawford A., Czuczka T. (2013). *Angela Merkel: A Chancellorship Forged in Crisis*.

[17] Hebel M. (2018). *Merkel: Bilanz und Erbe der Kanzlerschaft*.

[18] Heckel M. (2009). *So regiert die Kanzlerin: Eine Reportage*.

[19] Horol’skaya, M. (2021), Leading German parties on the eve of elections to the Bundestag, *Mirovaya Ekonomika i Mezhdunarodnye Otnosheniya*, no. 9, pp. 25‒33.

[20] Knaus G. (2020). *Welche Grenzen brauchen wir?: Zwischen Empathie und Angst—Flucht, Migration und die Zukunft von Asyl*.

[21] Kokeev, A. (2021), Germany and Biden’s transatlantic strategy: Unresolved problems and new challenges, *Mirovaya Ekonomika i Mezhdunarodnye Otnosheniya*, No. 9, pp. 56–68.

[22] Kornelius S. (2013). *Angela Merkel: Die Kanzlerin und ihre Welt*.

[23] Languth, G. (2005), *A. Merkel: Aufstieg zur Macht,* München.

[24] Levitsky S., Ziblatt D. (2018). *Wie Demokratien sterben: Und was wir dagegen tun können*.

[25] Mishra R. (2010). *Angela Merkel—Machtworte: Die Standpunkte der Kanzlerin*.

[26] Münkler H., Münkler M. (2016). *Die neuen Deutschen: Ein Land von seiner Zukunft*.

[27] Mut zu Zwischentönen (2000), *Spiegel*, Dec. 24. https://www.spiegel.de/politik/mut-zu-zwischentoenen- a-c409d19f-0002-0001-0000-000018124491. Cited November 15, 2021.

[28] Pavlov, N.V. (2018a), Merkel 4.0 is a reality, *Historia Provinciae—Zhurnal Regional’noj Istorii*, vol. 2, no. 2, pp. 80‒90.

[29] Pavlov N.V. (2018). *Vneshnyaya politika kanclera A. Merkel’ (2005‒2017)*.

[30] Pavlov, N.V. (2019), Germany after Merkel (Should we expect changes in the foreign policy of Germany?), *Polis.**Politicheskie Issledovaniya,* No. 6, pp. 22–35.

[31] Qvortrup M. (2017). *Angela Merkel: Europe`s Most Influential Leader*.

[32] Renterghem M. (2016). *C`etait Merkel*.

[33] Resing V. (2017). *A. Merkel—Die Protestantin: Ihr Aufstieg, ihre Krisen—und jetzt?*.

[34] Schramm Ju. (2021). *Fifty Shades of Merkel*.

[35] Schumacher H. (2006). *Die zwölf Gesetze der Macht: Angela Merkels Erfolgsgeheimnisse*.

[36] *Sovremennaya Germaniya: ekonomika i politika* [Modern Germany: Economics and Politics] (2015), Ed. by V.B. Belov, Moscow: IE RAN, Ves’ Mir.

[37] Stock, W.A. (2005), *Merkel: Eine politische Biographie,* München: Olzog.

[38] Timofeev, P. and Horol’skaya, M. (2021), COVID-19 pandemic as a challenge to Franco–German leadership in the EU, *Mirovaya Ekonomika i Mezhdunarodnye Otno-sheniya*, vol. 65, no. 8, pp. 72–80.

[39] Timoshenkova, E.P. (2020), The German party political system during Angela Merkel‘s Chancellorship (2005‒2017), in *Doklady Instituta Evropy*, No. 369, Moscow: IE RAN. 10.15211/report22020_369.

[40] Timoshenkova, E.P. (2021), “Formula of Power” of Chancellor A. Merkel: Formation (2005‒2013), *Sovremennaya Evropa,* no. 5. pp. 161‒171. 10.15211/soveurope52021161171

[41] Ulrich, B. (2017), Angela Merkel Neudefinition von Führungsstärke, *Strategic Europe*, Sep. 22, 2017. https://carnegieeurope.eu/2017/09/22/de-pub-73198. Cited July 20, 2021.

[42] Vasil’ev, V. (2021), Germany and the European Union after the era of chancellor A. Merkel, *Mirovaya Ekonomika i Mezhdunarodnye Otnosheniya*, no. 9, pp. 43–55.

[43] Vasil’ev, V.I. (2018), “Mercalization” of the SPD: A departure from the “eastern policy” of W. Brandt, *Mezhdunarodnaya Zhizn’,* no. 9, pp. 47‒64.

